# Empathy in Adults with Acquired Brain Injury: a Systematic Review and Meta-Analysis

**DOI:** 10.1007/s11065-025-09667-5

**Published:** 2025-06-18

**Authors:** Emily Clements, Kristin Naragon-Gainey, Michael Weinborn, Carmela Pestell, Dawn Neumann, David Preece, Rodrigo Becerra

**Affiliations:** 1 School of Psychological Science, The University of Western Australia, Perth, Australia; 2 Morsani College of Medicine, University of South Florida, Tampa, FL USA; 3 School of Psychology and Speech Pathology, Curtin University, Perth, Australia; 4 Department of Psychology, Stanford University, Stanford, CA USA

**Keywords:** Acquired brain injury, Empathy, Cognitive, Affective, Social cognition

## Abstract

Empathy is the ability to recognise, share and understand others’ emotional states. Increasing evidence suggests that empathy may be impacted by acquired brain injury (ABI), with consequences for social and emotional functioning. However, the literature has been characterised by inconsistent findings and small sample sizes. To address these limitations, we provide the first meta-analytic review of empathy in adults with ABI. Specifically, the review aimed to quantify the degree of impairment in adults with ABI across four empathy-related domains: cognitive, affective, empathic concern (e.g. sympathy) and personal distress. We also sought to estimate the prevalence of deficits in each area and explore whether demographic and injury factors moderate impairment. A systematic search yielded 29 studies measuring self-reported empathy in adults with ABI versus healthy, matched peers. A series of random-effects meta-analyses revealed moderate deficits in cognitive empathy (Hedges’ *g* =  − 0.68, 95% CI [− 0.87, − 0.50]) and affective empathy (Hedges’ *g* =  − 0.43, 95% CI [− 0.65, − 0.21]), as well as small-to-moderate deficits in empathic concern (Hedges’ *g* =  − 0.38, 95% CI [− 0.63, − 0.13]). No significant difference was found for personal distress. We estimated the proportion of ABI participants scoring equal to or more than 1 SD below the normative mean to be 15.3–35.0%, depending on the empathy subcomponent. Our results highlight that empathy deficits may play an important role in functional or emotional difficulties post-brain injury. This demonstrates the need for routine clinical assessment of empathy in survivors of brain injury and the need to develop interventions which target both cognitive and affective components.

**Supplementary Information:**

The online version contains supplementary material available at 10.1007/s11065-025-09667-5.

## Introduction

Acquired brain injury (ABI) is recognised as a leading cause of death and disability worldwide (Feigin et al., 2021; Maas et al., 2008). The term ABI refers to any brain injury incurred after birth, excluding degenerative disease (Ciuffreda & Kapoor, 2012). Its aetiology is highly heterogeneous, comprising traumatic brain injury (TBI, i.e. injury caused by external force to the head) and non-TBI, such as cerebral vascular accident, aneurysm, brain tumour and hypoxia. Among the many challenges faced by survivors, the loss of empathy is particularly distressing for patients and their families (Hillis, 2020; Wood & Williams, 2008). Empathy is a core social competency: the ability to share and understand others’ emotions permits social bonding and responsivity to others’ needs (de Vignemont & Singer, 2006; Decety et al., 2016). When these skills are lacking, survivors of brain injury often struggle to maintain interpersonal relationships, resulting in social isolation and poor mental health (Salas et al., 2018; Williams et al., 2020). Despite such well-documented impacts, little is known about the nature of empathy deficits after ABI. Prevalence estimates vary widely (e.g. de Sousa et al., 2012; Osborne-Crowley et al., 2020), and the magnitude of the effects is yet to be estimated with precision. Moreover, it is unclear how the various components of empathy may be relatively affected following ABI. Therefore, the current study aimed to estimate the prevalence and characteristics of empathy deficits in individuals with ABI via meta-analysis.

### Empathy

Although definitions vary, there is a developing consensus that empathy denotes recognising, sharing and understanding another’s emotional experiences (Eklund & Meranius, 2021). In addition, empathy requires an awareness that the emotional source originates outside oneself (de Vignemont & Singer, 2006). Thus, empathy is considered a multicomponent phenomenon (Weisz & Cikara, 2021). While empathy is often characterised as a personality trait, with moderate to high degree of stability over time (Fassbender et al., 2022; Grühn et al., 2008), others acknowledge there is some variability influenced by context, such as situational, relational and motivational factors (Cuff et al., 2016). Women tend to self-report higher levels of empathy (Baez et al., 2017), which has been attributed to gender role stereotypes and an evolutionary history of maternal care (Rochat, 2023).

Numerous theoretical models have attempted to characterize the nature of empathy. Among these, there is generally agreement that empathy consists of both affective and cognitive processes (Decety & Jackson, 2004; Thompson et al., 2019; Zaki & Ochsner, 2012). Affective empathy refers to viscerally sharing another’s emotion, whilst cognitive empathy denotes the ability to recognise and understand that emotion (Decety & Jackson, 2004). Increasing evidence suggests that these are separable processes (Kogler et al., 2020; Uribe et al., 2019), although they appear to be largely coactivated in real-world social situations (Depow et al., 2021; Löchner et al., 2023). Affective empathy is thought to primarily occur via automatic mechanisms, including perception (e.g. recognising facial expressions) and mimicry/embodiment (e.g. spontaneous facial mimicry of another’s emotional expression; Preston & de Waal, 2002; Rymarczyk et al., 2016). Conversely, cognitive empathy likely reflects higher-level effortful processing, permitting mentalizing, perspective-taking and self-other distinction (Shamay-Tsoory et al., 2009; Thompson et al., 2019). Thus, empathy can be conceptualised as a set of distinct yet overlapping processes that unfold over the course of an interaction between a target (e.g. the person experiencing the emotions) and an observer.

Several empathy models have also highlighted the importance of emotion regulation (e.g. Davis, 1980; Decety & Jackson, 2004; Thompson et al., 2019), since emotions evoked by empathy are subject to emotion regulatory processes. Broadly, emotion regulation refers to the processes through which one manages the experience and expression of emotion, through the use of various strategies such as attentional deployment and cognitive reappraisal (Gross, 2015). Research shows that emotion regulation ability may determine whether empathic arousal (e.g. the shared emotional state induced by witnessing another’s emotions) leads to *empathic concern* or *personal distress*, with consequences for social behaviour and emotional wellbeing. Specifically, well-regulated empathic arousal is thought to result in empathic concern (i.e., feelings of sympathy and motivation to care; Decety et al., 2016), which in turn increases the likelihood of prosocial behaviour and sensitive responding (Brethel-Haurwitz et al., 2020). Conversely, poorly regulated empathic arousal may be more likely to lead to personal distress (Eisenberg & Eggum, 2009), a self-focused state of negative arousal consistently linked to avoidance. Traditionally, empathic concern and personal distress have been considered core aspects of empathy and are included in some commonly used psychometric tools; however, more recent frameworks have regarded these as outcomes, distinct from empathy (Brett et al., 2023; Eisenberg & Eggum, 2009; Klimecki & Singer, 2013; Thompson et al., 2019). Nonetheless, these related constructs may have specific relevance for ABI, since emotion regulation is often disrupted by brain injury. As such, they will be included in this analysis.

### Empathy After Acquired Brain Injury

Several brain regions implicated in empathic processing are vulnerable to brain injury, namely ventromedial prefrontal areas, as well as right cortical and limbic regions (Leigh et al., 2013; Shamay-Tsoory et al., 2004). As such, empathy deficits are common, enduring sequelae of ABI (Williams et al., 2020). To date, most empathy research has been conducted on participants with TBI. The most consistent finding in the literature is that adults with moderate-to-severe TBI report low affective empathy compared to healthy, matched controls (e.g. de Sousa et al., 2010; Neumann et al., 2014; Williams & Wood, 2010). However, several studies have not found significant differences (Driscoll & Krueger, 2012; Nijsse et al., 2019a; Osborne-Crowley et al., 2020), which researchers have attributed to participants’ lesion location and potentially limited insight. There is also some evidence that individuals with TBI experience impaired cognitive empathy relative to controls (de Sousa et al., 2010; Neumann et al., 2014; Spikman et al., 2012). Low empathy has also been documented in other forms of ABI, including low-grade glioma (Herbet et al., 2015), subarachnoid haemorrhage (Brand et al., 2015; Buunk et al., 2017) and stroke (Hillis, 2014).

### Limitations in the Literature

Despite empathy deficits being reported across diverse forms of ABI, there is much that remains unknown about empathic functioning in this population. Firstly, the prevalence of empathy deficits in ABI is yet to be precisely estimated. Even within TBI samples, reported prevalence rates vary widely. For example, low affective empathy is reported to occur in 23–71% of TBI patients (versus 14–34% matched controls; de Sousa et al., 2012; Osborne-Crowley et al., 2020; Williams & Wood, 2010), with estimates of cognitive empathy deficits ranging from 34 to 50% (versus 18% controls; de Sousa et al., 2010; Zupan et al., 2018). This likely reflects the significant heterogeneity present within TBI (Covington & Duff, 2021), as well as the constraints of small sample sizes.

Secondly, it is unclear how the components of empathy and its outcomes (i.e. empathic concern, personal distress) may be affected after ABI. Much ABI literature has only examined a single dimension of empathy, despite contemporary theoretical models emphasizing both cognitive and affective components (e.g. Bird & Viding, 2014). This is inconsistent with other clinical research, which has documented selective deficits (e.g. cognitive and/or affective) across a variety of conditions, including schizophrenia (Horan et al., 2015)*,* autism spectrum disorder (Song et al., 2019), eating disorders (Kerr-Gaffney et al., 2019) and neurodegenerative conditions (Pick et al., 2019). To confuse matters further, empathy components have often been conflated in the literature, limiting comparison and interpretation of results. For example, it has been common practice to use measures of empathic concern and/or personal distress to estimate affective empathy (e.g., de Sousa et al., 2010; Shamay-Tsoory et al., 2009). However, these are increasingly recognised as conceptually distinct phenomena, with discrete neural substrates (Decety, 2010; Singer & Lamm, 2009). As discussed earlier, affective empathy is increasingly defined as the perceptual and mimicry processes that allow us to share another’s emotion. On the other hand, empathic concern is feeling *for* another (e.g. sympathy) and contains a motivational component to help, whilst personal distress is characterised by aversive, negative arousal. Thus, empathic concern and personal distress are end states that may occur downstream of empathy, depending on the individual’s ability to regulate their empathic arousal. It is important to dissociate these aspects, since empathy components may contribute differently to socioemotional outcomes (Weisz & Cikara, 2021) and would likely require different treatment approaches.

Thirdly, there is emerging evidence that the typically observed female advantage for empathy may be altered after brain injury. Recently, Zupan et al. (2018) found no sex differences in self-rated empathic concern or perspective-taking in a large sample of adults with TBI. However, other research has found higher affective empathy in women versus men after TBI (Williams & Wood, 2010; Wood & Williams, 2008) and higher overall empathy in females versus males with low-grade glioma (Herbet et al., 2015). These equivocal findings merit further attention.

Lastly, it would be beneficial to clarify how broader demographic (in addition to sex) and clinical characteristics may impact empathic functioning. Given the significant heterogeneity associated with ABI, it is important to identify the factors that underlie observed differences*.* Past research suggests that self-reported empathy is unrelated to general cognitive functioning, injury severity or time since injury (Neumann et al., 2014; Osborne-Crowley et al., 2020; Williams & Wood, 2010), although these studies were limited to TBI samples and may have lacked power to detect an effect. On the other hand, meta-analyses have reported relationships between demographic and brain injury variables and other aspects of socio-emotional cognition. For example, theory of mind impairments has been linked to greater injury severity, older age and fewer years of education in TBI and stroke populations (Lin et al., 2021a; Nijsse et al., 2019b). Furthermore, injury type has been found to moderate alexithymia (e.g. difficulty identifying and describing emotions), with TBI patients significantly more impaired than participants with other forms of brain injury (Fynn et al., 2021). Since research has found close relationships between alexithymia, theory of mind and empathy (e.g. Di Tella et al., 2024; Schurz et al., 2021), this area should be explored further.

### Current Study

The present meta-analysis aims to provide the first comprehensive account of empathic functioning after ABI, by (1) quantifying the extent to which self-reported empathy and its outcomes (e.g. empathic concern, personal distress) differ between adults with ABI and healthy controls and (2) estimating the prevalence of self-reported empathy deficits in an ABI population. Finally, since research reports mixed findings for sex differences in community versus brain-injured samples, this study aims to (3) determine any sex differences in empathic functioning after ABI. Where possible, additional demographic (e.g. age and years of education) and injury-related factors (e.g. time since injury, injury type, severity and lesion location) known to impact social cognition will be explored as predictors of empathy impairment.

## Method

This meta-analysis followed the guidelines of Preferred Reporting Items for Systematic Reviews and Meta-Analyses 2020 (Page et al., 2021). The review was registered prospectively on Inplasy (available from https://inplasy.com/inplasy-2022-11-0125/).

### Literature Search

A systematic literature search was conducted on 15 March, 2024 to identify studies measuring self-reported empathy in adults with ABI*.* The search strategy was adapted from similar reviews (e.g. Fynn et al., 2021) and developed in consultation with a research librarian. Five electronic databases (Ovid MEDLINE, ProQuest, PsycINFO, Scopus and Web of Science) were searched using the following terms: (empath* OR interpersonal reactivity OR emotion* contagion OR experience sharing OR theory of mind OR mentali?ing OR perspective taking OR social cogniti*) AND ((brain OR head OR craniocerebral OR cranial OR cerebr*)Adj2(injur* OR trauma* OR incident OR accident OR damage OR concussi* OR brain ischemi* OR stroke OR diffuse axonal injur* OR tbi OR abi OR abd)). Details of the search strategy used in each database is available in the Supplementary Material (Table S1). No limitations were placed on publication date, and grey literature was included to reduce the risk of publication bias (Higgins et al., 2024)*.* Finally, reference sections of included studies were hand-searched to identify relevant papers.

### Study Selection

Covidence software was used to manage all stages of the data screening and extraction process. Studies were eligible for inclusion if they met the following criteria: (1) full-text availability in the English language; (2) data available for individuals diagnosed with ABI; (3) presence of a comparator group of non-brain-injured individuals; (4) adult population (mean age 18–70 years) and (5) assessment of empathy using any validated self-report measure. The age limit of 70 years was chosen to reduce the risk of sampling participants with undetected dementia. Studies were excluded if participants were diagnosed with neurodegenerative disease (e.g. multiple sclerosis), as this does not align with our target definition of ABI. Qualitative studies, case studies and review articles were also excluded. After removal of duplicates, titles and abstracts were screened by the first author (EC) against the inclusion and exclusion criteria outlined above, then full text screened. In addition, two doctoral students (AB, AH) with undergraduate training in psychology independently evaluated a total of 40% of the records at each stage of the screening, with conflicts resolved via discussion and consensus.

### Extracted Data

Data were manually extracted by one author (EC) and checked by a second author (RB). Extracted data included the study aims, funding sources and any reported conflicts of interest, participant recruitment details, comparator group characteristics (e.g., age, sex) and mean (SD) empathy levels for the ABI sample and comparator group (including total scores and/or subscales). Where studies included multiple ABI subgroups, the means and SDs of empathy scores of each subgroup were pooled to produce a single estimate. Prevalence data for low empathy was also extracted for ABI and control groups (i.e. the proportion of empathy scores equal to or more than 1 SD below the mean on published norms for each measure). Each sample contributed only one effect size per construct, in order to ensure statistical independence. If studies listed multiple effect sizes for a single construct, we calculated an average effect size. According to the recommendations of Borenstein et al. (2009), we accounted for the correlation between scales when computing the variance for each mean effect size.

The items of each empathy measure were examined against our operational definitions of empathy. The decision to link measures to different empathy components was made via consensus between three authors (EC, RB, DP). Affective empathy was defined as emotion sharing and cognitive empathy as the process of recognising and understanding another’s emotions. Although it has been common practice for Interpersonal Reactivity Index (IRI) to sum the *perspective-taking* and *fantasy* subscales as a proxy of cognitive empathy (e.g. Shamay-Tsoory et al., 2009; Yeh et al., 2015), the psychometric validity of this approach has been questioned (Wang et al., 2020) and the *fantasy* items do not align with commonly accepted definitions of cognitive empathy. Hence, we preferentially extracted the *perspective-taking* subscale score to estimate cognitive empathy, in line with the recommendations of Wang et al. (2020). Affective and cognitive empathy were conceptualised to combine to produce overall empathy ability (i.e. feeling, sharing and understanding another’s emotional experiences). Whilst several studies reported a total scale score for the IRI (e.g. overall empathy), we did not extract this for several reasons. Firstly, no items assess emotion sharing, limiting the construct validity of a total empathy score. Secondly, recent psychometric analyses have not supported the presence of a higher order empathy factor from the four IRI subscales (Raimondi et al., 2023; Wang et al., 2020), and IRI subscales are commonly reported to be inversely correlated (i.e. *personal distress* and *perspective-taking*, Chrysikou & Thompson, 2016; Davis, 1983). Empathic concern was defined as feeling sympathy and compassion for another and personal distress as an aversive state of negative arousal elicited by others’ distress (Davis, 1980).

### Risk of Bias Assessment

Study design quality was assessed using the Quality Assessment of Diagnostic Accuracy Studies-2 framework (QUADAS-2; Whiting et al., 2011). QUADAS-2 consists of four domains: (1) patient selection; (2) index test; (3) reference standard and (4) flow and timing. Two assessors (EC, AB) independently appraised each study for risk of bias and concerns about applicability to the review question, with studies being rated as low, unclear or high risk.

### Analytic Strategy

To estimate the extent to which self-reported empathy and its outcomes (e.g. empathic concern, personal distress) differ between adults with ABI and healthy controls (aim 1), a series of meta-analyses were performed using Comprehensive Meta-Analysis Version 4 (Borenstein et al., 2022). Studies were grouped for synthesis according to whether they measured overall empathy or an empathy component (cognitive, affective, empathic concern or personal distress), with separate meta-analyses conducted for each. Hedges’ *g* was used to estimate effect sizes and is reported with 95% confidence intervals (Hedges & Olkin, 1985). We chose to use a random-effects model (Cheung & Vijayakumar, 2016), which assumes that a distribution of true effect sizes exists in the population, rather than a single effect size. The magnitude of Hedges’ *g* effect sizes was interpreted according to Cohen’s (1988, 1992) guidelines of small (0.2), medium (0.5) and large (0.8).

### Heterogeneity in Effect Sizes

Heterogeneity in effect sizes was examined in four ways. First, the *Q*-statistic was calculated for each meta-analytic estimate, to test whether heterogeneity was statistically significant. A significant *p* value suggests that the true effect sizes vary. Since the *Q*-statistic is influenced by the number of included studies, we also examined *I*^2^, an estimate of the proportion of observed variance reflecting true differences in effect sizes (e.g., versus random error; Borenstein et al., 2009). Finally, we examined tau-squared (*τ*^2^) and 95% prediction intervals to estimate the magnitude of true dispersion in effect sizes. Tau-squared is the variance of true effects, whilst 95% prediction intervals represent the expected range of true effects in 95% of similar future studies (Borenstein et al., 2009).

### Pooled Prevalence

The second goal of this study was to estimate the prevalence of low empathy in ABI. Therefore, we calculated the proportion of participants (clinical and controls) who scored equal to or more than 1 SD below the mean on the empathy measure for each study (e.g. at or below the 16th percentile)*.* This criterion was chosen as it commonly designates ‘low’ or ‘moderately low’ empathy on self-report measures of empathy (e.g. Baron-Cohen & Wheelwright, 2004; Brett et al., 2023; Mehrabian, 1997). Mean scores and standard deviations were derived from the published norms for each measure or the control samples if no published norms were available. Due to low availability of prevalence data, we used additional simulated data to increase power and precision. Where prevalence data was unavailable, we simulated data in *RStudio* (2023) according to each study’s reported mean and standard deviation. We then identified the proportion of cases associated with a score of ≥ 1 SD below the mean for each sample of simulated data and combined these in a random-effects meta-analysis model. To validate this method, raw empathy data were examined for normality according to the criteria outlined by Curran et al., (1996; i.e., skew < 2; kurtosis < 9) and paired-samples *t* tests were conducted to compare real versus simulated data (Kleijnen, 1999). Similar approaches have previously been used to estimate the prevalence of emotional processing difficulties in ABI populations (Babbage et al., 2011; Fynn et al., 2021).

### Moderator Analysis

To explore sex differences (aim 3) in empathic functioning after ABI, the effect of sex (% male) on meta-analytic effect sizes was tested via the meta-regression function in comprehensive meta-analysis.

Meta-regression tests the relationship between the study moderator and empathy differences, using the random effects framework. However, sex effects can be difficult to identify via meta-regression when there is a lack of variability in sex composition across studies (Craig Aulisi et al., 2023). To address this issue, we entered empathy data for males and females separately (where available) to increase power to detect sex as a moderator.

Where significant heterogeneity among empathy effect sizes was found, additional demographic factors and injury characteristics were explored as moderators, since these have been shown to impact the degree of cognitive impairment after brain injury (Rabinowitz et al., 2018; Spikman et al., 2012). Continuous moderators (age, injury severity, time since injury) were tested via meta-regression, providing there were four or more studies reporting moderator data. Injury severity was measured using the Glasgow Coma Scale (GCS; Teasdale & Jennett, 1974) and post-traumatic amnesia (PTA), commonly used clinical metrics of brain injury severity (Perrin et al., 2015). A statistically significant regression coefficient would support the hypothesis that these variables moderate empathy differences in ABI versus controls. Categorical moderators (e.g. injury type, injury location, measure type) were tested using a subgroup analysis within a random-effects model to account for study-level variability. Differences between subgroups were tested using the fixed-effect model to assess the influence of moderators on effect sizes, and Cochran’s *Q* test was used to determine whether the moderator categories (e.g. different injury types) differ in their impact on the outcome.

### Publication Bias

Several methods were used to evaluate whether publication bias may have affected the current study. Symmetry of funnel plots (Sterne et al., 2011) was examined both visually and via Egger’s intercept (Egger et al., 1997), where the latter tests a null hypothesis suggesting an absence of publication bias. We also report Rosenthal’s (1979) fail-safe *Ns*, which represent the number of unpublished studies with an average effect size of zero that would be required to nullify the significance of the observed effect size. Generally, fail-safe *Ns* larger than 5*n* + 10 are considered robust to publication bias (*n* = number of effect sizes in the sample; Rosenthal, 1991).

## Results

### Screening Results

**Fig. 1:**
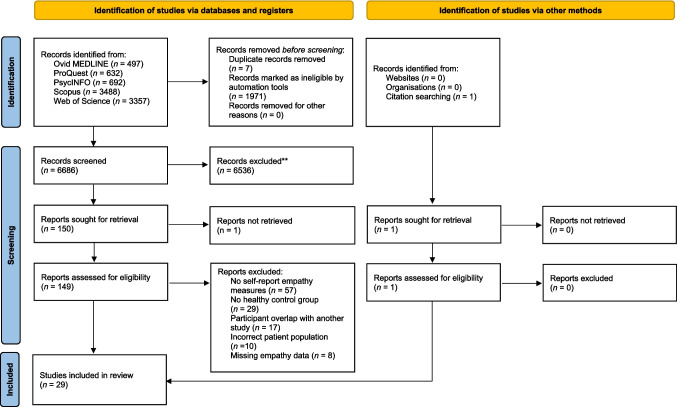
Preferred reporting items for systematic reviews and meta-analyses 2020 study selection process (Page et al., 2021)

The PRISMA flowchart detailing the study selection process is shown in Fig. 1*.* Interrater reliability for study screening was high (kappa = 0.83), and 100% agreement was reached by consensus for study extraction. We contacted 38 study authors to clarify uncertainties or obtain additional data. Furthermore, all retrieved studies were checked to ensure independence of samples and to avoid double counting data (Lipsey & Wilson, 2001)*.* In total, 17 duplicate samples were identified and excluded. One additional study was identified by searching the reference lists of eligible records, resulting in a total of 29 included studies.

### Study Characteristics

Characteristics of the included studies are displayed in Table 1. Most studies were peer-reviewed publications, with the addition of three postgraduate theses (no. 5, no. 14; no. 19). The retrieved studies measured empathy in patients with TBI (*k* = 12), stroke (*k* = 6), ABI (mixed aetiology; *k* = 6), brain tumour (*k* = 3) and lesions resulting from surgical excision or resection for treatment of epilepsy (*k* = 2). Participants sustained right hemisphere lesions (*n* = 255), left hemisphere lesions (*n* = 240) and bilateral injuries (*n* = 40), with laterality unspecified in the remainder (*n* = 818). The mean age of participants ranged from 30.1 to 67.7 years and males were overrepresented across studies (*M* % males = 68.16). Injury severity was mostly obtained from TBI studies and ranged from mild to severe, although 14 studies did not report this data. Sample sizes of ABI patients ranged from 11 to 192 (*M* = 46.66, *SD* = 40.58) and mean time since injury also varied widely (13.64 months to 30 years). Studies were conducted in the UK (*k* = 5), US (*k* = 5), Canada (*k* = 4), Germany (*k* = 4), Australia (*k* = 3), Netherlands (*k* = 2), Taiwan (*k* = 2), Israel (*k* = 2), France (*k* = 1) and China (*k* = 1).

**Table 1:** Characteristics of samples included in the meta-analysis

No	Author (year)	ABI type	*n*	% male	Mean age (SD)	Mean years of education (SD)	Injury location	Injury severity	Mean time since injury (SD)	Empathy measure	Mean empathy TS (SD)	Mean cognitive empathy (SD)	Mean affective empathy (SD)	Mean empathic concern (SD)	Mean personal distress (SD)
1	Adams et al. (2021)	Stroke	35	65.70	64.49 (12.92)	14.09 (3.72)	RH (*n* = 18); LH (*n* = 17)	-	13.64 (2.29)^c^	IRI	-	17.76 (5.15) PT	-	21.06 (3.94) EC	-
2	Boucher et al. (2015)	Surgical resection	45	43.33	38.15 (9.34)	13.05 (2.71)	anterior temporal lobe (*n* = 15); insula (*n* = 15)	-	3.95 (3.24)^d^	IRI	-	16.40 (5.34) PT	-	19.65 (4.60) EC	9.8 (6.05) PD
3	Bramham et al. (2009)	ABI	34	47.06	42.82 (14.65)	-	RH (*n* = 15); LH (*n* = 14); bilateral (*n* = 6)	-	7.14 (6.29)^d^	SEQ (empathy subscale)	-	-	-	20.65 (3.33)	-
4	Chen et al. (2016)	Tumour	34	52.94	38.50 (7.04)	9.12 (2.10)	Insular: RH (*n* = 7); LH (*n* = 10); posterior: RH (*n* = 8); LH (*n* = 9)	-	-	IRI-C (Chinese)	-	9.94 (2.53) PT	-	12.56 (3.20) EC	9.00(-) PD
5	Darling (2001)	ABI	20	70.00	36.70 (10.44)	13 (-)	-	-	6.03(-)^d^	HES, QMEE	-	33.55 (5.83) HES	23.70 (27.65) QMEE	-	-
6	Driscoll and Krueger (2012)	TBI	192	100.00	58.30 (3.10)	14.80 (2.50)	-	-	30(-)^d^	BEES	-	-	29.20 (26.40)	-	-
7	Gallant and Good (2020)	mTBI	32	16.90	19.88 (2.42)	-	-	Mild	-	IRI, QCAE	59.13 (12.04) QCAE	33.50 (8.45) (QCAE cognitive) 19.16 (4.37) PT	25.63 (5.81) (QCAE affective)	20.47 (4.30) EC	12.38 (5.21) PD
8	Goebel et al. (2018)	Tumour	30	43.00	46.77 (14.19)	13.20 (2.07)	RH (*n* = 12); LH (*n* = 12); infratentorial (*n* = 6)	-	-	EQ	40.50 (10.39)	-	-	-	-
9	Grattan and Eslinger (1989)	ABI	50	62.00	56.69 (18.79)	12.14 (2.42)	LH (*n* = 10); RH (*n* = 28), bilateral (*n* = 2)	-	-	HES	-	27.16 (4.62)	-	-	-
10	Holtmann et al. (2023)	Stroke	20	70.00	59.5 (7.5)	11.4 (1.8)	LH (*n* = 13). RH (*n* = 7)	-	5.56 (4.32)^d^	IRI (German)	-	13.92 (0.52) PT	-	13.78 (0.38) EC	9.73 (0.58) PD
11	Kgolo et al. (2021)	ABI	42	45.24	51.70 (15.04)	13.26 (3.55)	Frontal: LH (*n* = 12); RH (*n* = 6) non-frontal: LH (*n* = 12); RH (*n* = 9); bilateral (*n* = 3)	-	-	EQ	-	5.04 (2.93) EQ cognitive	5.00 (2.38) EQ affective	-	-
12	McDonald et al. (2017)	TBI	30	83.33	47.27 (14.64)	13.60 (2.64)	LH (*n* = 10); RH (*n* = 6); bilateral (*n* = 9); other (*n* = 1); not available (*n* = 4)	Severe (mean PTA 51.37 (44.87)^a^	13.40 (13.40)^d^	BEES, IRI	-	17.52 (5.23) PT	34.13 (23.70) BEES	19.01 (4.25) EC	11.10 (5.87) PD
13	Milders et al. (2003)	TBI	17	59.00	30.5 (13.3)	11.6 (1.7)	-	Severe: mean PTA 33.6 (27)^a^; mean GCS 6.2 (2.6)	4.4 (4.9)^d^	EEQ	-	-	27.8 (28.7)	-	-
14	Minga (2014)	Stroke	11	45.45	52.8 (5.50)	-	RH (*n* = 11)	-	5.9^d^ (-)	TEQ	-	-	-	44.73 (9.33)	-
15	Muller et al. (2010)	TBI	15	86.67	37.20 (12.30)	10.40 (2.00)	RH (*n* = 3); LH (*n* = 6); bilateral (*n* = 6)	Severe. mean GCS 4.8 (1.7)	102.9 (121.2)^c^	IRI	-	14.93 (4.43) PT	-	18.80 (3.87) EC	14.53 (3.54) PD
16	Neumann et al. (2014)	TBI	60	62.00	40.98 (12.45)	-	-	Moderate–severe: mean GCS 4.47 (2.48); mean PTA 19.9 (38.16)	13.68 (10.54)^d^	IRI	-	14.77 (6.23) PT	-	19.85 (4.30) EC	-
17	Neumann et al. (2021)	TBI	105	57.14	39.84 (13.50)	14.15 (2.00)	-	Mild–severe	5.1 (median)^d^	IRI	-	17.25 (6.10) PT	-	20.63 (5.20) EC	-
18	Nijsse et al. (2019b)	Stroke	148	69.59	67.70 (11.20)	-	LH (*n* = 50); RH (*n* = 62); vertebrobasilar (*n* = 36)	No symptoms (*n* = 37), minor *(n* = 84), moderate *n* = 25), moderate–severe (*n* = 2)	3.7 (11.2)^d^	BEES (Dutch)	-	-	32.06 (22.50)	-	-
19	Paterson (2011)	TBI	19	78.95	45.00 (12.00)	11.60 (2.10)	-	Severe	129.1 (132.56)^c^	BEES, BES	67.47 (7.88) BES	31.95 (3.49) BES cognitive	35.53 (6.46) BES affective; *z* = − 0.48 (1.31) BEES	-	-
20	Pertz et al. (2021)	Tumour	43	51.12	64.05 (11.69)	14.92 (3.70)	-	-	-	IRI (German)	-	14.12 (2.64) PT	-	14.51 (2.75) EC	11.23 (3.34) PD
21	Pertz et al. (2022)	Stroke	36	66.67	57.90 (8.50)	14.10 (2.40)	RH (*n* = 13); LH (*n* = 19); bilateral (*n* = 4)	-	33.81 (24.42)^c^	IRI (German)	-	14.20 (2.90) PT	-	14.60 (2.10) EC	11.80 (2.50) PD
22	Sawczak et al. (2022)	Surgical excision	16	31.25	37.60 (11.70)	15.30 (2.30)	LH (*n* = 8); RH (*n* = 8)	-	-	TEQ	-	-	-	48.33 (6.21)	-
23	Shamay-Tsoory et al. (2004)	ABI	51	78.43	37.25 (14.09)	12.80 (1.92)	Prefrontal (*n* = 36); parietal(*n* = 15)	-	-	IRI, QMEE	-	*z* = −0.13 (0.47) IRI: (FS + PT)	*z* = 0.11 (0.43) QMEE	-	-
24	Shamay-Tsoory et al. (2009)	ABI	30	80.00	36.03 (15.04)	12.95 (2.09)	RH (*n* = 11); LH (*n* = 19); bilateral (*n* = 3)	-	8.03 (8.43)^d^	IRI	-	1.54^e^ (5.19) PT	-	3.19^e^ (4.17) EC	− 1.08^e^ (5.25) PD
25	Spikman et al. (2012)	TBI	28	71.40	30.10 (12.90)	-	OFC (*n* = 15); DFC (*n* = 11); non-frontal (*n* = 19)	Moderate–severe: mean PTA 41(42)^a^; mean GCS 9.5(3.6)	35 (69)^c^	EEQ	-	-	13.10 (25.20)	-	-
26	Williams and Wood (2010)	TBI	64	82.80	35.84 (13.33)	12.14 (2.18)	-	Moderate–severe: mean PTA 16.85 (27.84), mean GCS 9.30 (4.46)	3.19 (2.58)^d^	BEES	-	-	15.08 (24.33)	-	-
27	Wood and Williams (2008)	TBI	89	66.30	42.33 (11.84)	11.72 (1.99)	-	Mean PTA 13.99 (28.99)^a^ Mean GCS 10.28 (4.44)	3.72(3.81)^d^	BEES	-	-	14.5 (35.06)	-	-
28	Yeh and Tsai (2014)	Stroke	34	50.00	61.35 (11.5)	9.09 (4.21)	LH (*n* = 14); RH (*n* = 20)	-	19.52 (7.38)^c^	IRI (Chinese)	-	3.27 (1.03) PT	-	3.07 (0.87) EC	2.92 (1.23) PD
29	Yeh et al. (2015)	TBI	23	60.87	32.04 (12.24)	12.74 (3.09)	RH (*n* = 11); LH (*n* = 5); bilateral (*n* = 7)	-	457.17 (333.41)^a^	IRI (Chinese)	-	2.32 (0.70) PT	-	3.26 (0.68) EC	3.04 (0.83) PD

*n* number of participants, *ABI* acquired brain injury, *TBI* traumatic brain injury, *mTBI* mild traumatic brain injury, *RH* Right hemisphere lesion, *LH* left hemisphere lesion, *OFC* orbitofrontal cortex, *DFC* dorsofrontal cortex, *PTA* post-traumatic amnesia, *GCS* Glasgow Coma Scale, *IRI* Interpersonal Reactivity Index, *SEQ* Social Emotional Questionnaire, *HES* Hogan Empathy Scale, *BAFQ* Brock Adaptive Functioning Questionnaire, *QMEE* Questionnaire Measure of Emotional Empathy, *BEES* Balanced Emotional Empathy Scale, *QCAE* Questionnaire of Cognitive and Affective Empathy, *EQ* Empathy Quotient, *EEQ* Emotional Empathy Questionnaire, *TEQ* Toronto Empathy Questionnaire, *BES* Basic Empathy Scale, *PT* Perspective-Taking subscale (IRI), *FS* Fantasy subscale (IRI), *EC* Empathic Concern subscale (IRI), *PD* Personal Distress subscale (IRI)

^a^Days

^b^Weeks

^c^Months

^d^Years

^e^Scores transformed on a scale from − 14 to + 14

### Empathy Measurement

A range of self-report empathy measures were used, with the most common being the Interpersonal Reactivity Index (IRI; Davis, 1980; *n* = 14). The IRI is a multidimensional self-report measure of empathy yielding four subscales: *perspective-taking* (e.g., “I try to look at everybody’s side of a disagreement before I make a decision”), *fantasy* (e.g., “I daydream and fantasize, with some regularity, about things that might happen to me”), *empathic concern* (e.g., “I often have tender, concerned feelings for people less fortunate than me”) and *personal distress* (e.g., “Being in a tense emotional situation scares me”). The Balanced Emotional Empathy Scale (BEES; Mehrabian, 1997; *n* = 6) also featured prominently, as well as its older version, the Questionnaire Method of Emotional Empathy (QMEE; Mehrabian & Epstein, 1972, also known as the Emotional Empathy Questionnaire [EEQ]; *n* = 4). The latter scales are designed to provide unipolar assessment of affective empathy. Additional measures included the empathy subscale of the Social Emotional Questionnaire (Bramham et al., 2009), Questionnaire of Cognitive and Affective Empathy (Reniers et al., 2011), Basic Empathy Scale (Jolliffe & Farrington, 2006), Toronto Empathy Questionnaire (Spreng et al., 2009), Empathy Quotient (Baron-Cohen & Wheelwright, 2004) and Hogan Empathy Scale (Hogan, 1969). Table S2 (Supplementary data) displays the empathy measures used and their corresponding component/s of empathy.

### Risk of Bias

The risk of bias assessment is displayed in Table S3 and graphically represented in Fig. S1. Interrater agreement was substantial (kappa = 0.78). Regarding potential bias in patient selection, only three studies were identified as enrolling a consecutive or random sample of patients (no. 2, no. 3, no. 25). Additionally, four studies were judged to have unclear inclusion criteria (no. 9, no. 13, no. 15, no. 29), and five studies contained clinical samples that differed from controls on one or more demographic variables (e.g., sex, age or years of education; no. 5, no. 14, no. 16, no. 17, no. 24). In the index test domain, 27 of 29 studies were judged to assess empathy in a standard, valid and reliable way. Of the two studies in this domain judged as ‘unclear’, one study developed its own questionnaire (no. 3), although the authors did report some validity measures. The other study used a non-standard approach to scoring the IRI, which may lack psychometric validity (Chrysikou & Thompson, 2016; Wang et al., 2020). Most studies were judged as likely to classify the target condition correctly using the reference standard (e.g. clinical diagnosis), with the exception of one study which relied on self-reported brain injury (no. 7). Concerning the flow and timing domain, it was unclear in five studies whether all patients received a medically confirmed diagnosis of their injury (no. 5, no. 12, no. 13, no. 16, no. 19), although in most cases, this affected only a small proportion of each sample. Additionally, several studies made participant exclusions after data collection (no. 2, no. 22, no. 24). Due to the broad nature of the review question, no studies were judged to have high concerns for applicability to the review question.

### Meta-Analytic Estimation of Effect Size

The results of the meta-analytic estimates for each empathy component are displayed in Table 2, with forest plots available in the Supplementary material (Fig. S2). The number of effect sizes for overall empathy (3) was deemed to be too few for meaningful synthesis; hence, one study that only included the total score (Goebel et al., 2018; *g* =  − 0.31, *p* = 0.23, 95%CI [− 0.81, 0.19]) was omitted from the meta-analyses. Compared to healthy controls, ABI participants self-reported significantly lower mean levels of affective empathy (*g* =  − 0.43, *p* < 0.001, 95%CI [− 0.65, 0.21]), cognitive empathy (*g* =  − 0.68, *p* < 0.001, 95%CI [− 0.87, − 0.50]) and empathic concern [*g* =  − 0.38, *p* < 0.01, 95%CI [− 0.63, − 0.13]). That is, individuals with ABI on average have empathic abilities or tendencies that are − 0.38 to − 0.68 *SD* below their non-brain-injured peers. However, there was no significant difference in personal distress self-reported by ABI participants versus controls. According to Cohen’s (1988, 1992) guidelines, these effects may be considered small-to-medium. The effect size for cognitive empathy was significantly larger than those of affective empathy, empathic concern and personal distress, as evidenced by less than 50% overlap in the respective 95% confidence intervals (Cumming & Finch, 2005). No other effect sizes differed significantly from each other. There was a high level of heterogeneity across meta-analytic estimates of effect size, with all *Q* statistics significant and 95% prediction intervals suggesting significant dispersion in true effects. This was most pronounced for personal distress, where the prediction interval indicated that the true effect would likely range from a large negative effect (*g* = −1.62) to a positive effect (*g* = 1.45) in 95% of similar future studies. Moreover, all 95% prediction intervals crossed the line of no effect, suggesting that individuals with ABI may score no differently or even higher than controls in some settings.

**Table 2:** Meta-analytic estimates of effect size for empathy components

	*N*	*k*	*g*	95%*CI*	*95% PI*	*z*	*τ*^*2*^	*Q(df)*	*I*^*2*^	FSN	Egger’s intercept
Affective empathy	1217	12	− 0.43***	− 0.65, − 0.21	− 1.17, 0.31	− 3.84	0.10	34.10(11)***	67.74	125	− 2.16
Cognitive empathy	1393	19	− 0.68***	− 0.86, − 0.49	− 1.40, 0.04	− 7.06	0.11	50.86(18)***	64.61	654	− 3.19
Empathic concern	1199	17	− 0.38**	− 0.63, − 0.13	− 1.38, 0.62	− 2.95	0.20	69.87(16)***	77.10	126	− 3.04
Personal distress	613	10	− 0.08	− 0.51, 0.35	− 1.62, 1.45	− 0.37	0.40	59.82(9)***	84.96	0	− 8.55

Estimates calculated using the random effects model

*N* sample size, *k* number of studies, *g* Hedges’ *g*, *CI* confidence intervals, *PI* prediction intervals, *z* value, *τ*^*2*^ statistic, *Q* Cochrane’s *Q* statistic, *I*^*2*^ statistic, *FSN* Fail-Safe N

*p < 0.05; **p < 0.01; ***p < 0.001

## Prevalence Estimates of Low Empathy

Altogether, we were able to obtain or calculate prevalence data from nine studies (no. 1, no. 10, no. 12, no. 13, no. 16, no. 17, no. 20, no. 21, no. 22) with simulated data being used for the remainder. Prevalence estimates for low empathy in ABI and control participants are displayed in Table 3, ranging from 15 to 35% for the ABI groups and 11 to 19% for the control groups. Relative to controls, a significantly greater proportion of participants with ABI reported low cognitive empathy, affective empathy and empathic concern, based on less than 50% overlap in their corresponding 95% confidence intervals (Cumming & Finch, 2005). However, the proportion of ABI participants reporting low personal distress did not differ significantly from controls.

**Table 3:** Meta-analytic estimates of prevalence of low empathy

	Estimated prevalence	*95% CI*
Affective empathy		
ABI	35.0%	23%, 50%
Controls	19.7%	13%, 30%
Cognitive empathy		
ABI	31.4%	23%, 41%
Controls	11.8%	7%, 18%
Empathic concern		
ABI	21.6%	17%, 27%
Controls	14.8%	10%, 21%
Personal distress		
ABI	15.3%	9%, 24%
Controls	11.3%	6%, 20%

Low empathy defined as equal to or more than 1 SD below the mean

We next conducted sensitivity analyses to determine whether the inclusion of simulated data may have altered primary results and conclusions. Raw empathy data for the seven available datasets (no. 1, no. 10, no. 12, no. 13, no. 20, no. 21, no. 22) were found to be approximately normally distributed (skew =  − 1.18 to 0.75; kurtosis =  − 1.99 to 2.18). Paired-samples *t* tests revealed non-significant differences between simulated and reported data for both ABI (cognitive empathy *t*(159) = 0.86, *p* = 0.56.; affective empathy *t*(46) = 0.67, *p* = 0.51; empathic concern *t*(174) = 0.19, *p* = 0.85; personal distress *t*(126) =  − 0.05, *p* = 0.96) and control samples (cognitive empathy *t*(0.92) = 0.92, *p* = 0.36; affective empathy *t*(46) =  − 0.14, *p* = 0.89; empathic concern *t*(181) =  − 1.32, *p* = 0.14, personal distress *t*(131) =  − 0.30, *p* = 0.76).

### Meta-Regressions

A random effects meta-regression was performed for each empathy component to determine whether demographic, measurement and injury factors could explain some of the variance in empathy effect sizes. Neither sex (% male), age nor injury severity revealed statistically significant effects (see Table 4). However, time since injury was found to significantly moderate affective empathy, accounting for 68% of the variance in effect sizes (*R*^2^ = 0.68, *b* = 0.02, 95%CI [0.005, 0.044], *z* = 2.45, *p* = 0.01). This suggests that for ABI participants, each additional year post-injury is associated with 0.02 increase in Hedges’* g* for affective empathy. In addition, higher empathic concern scores were associated with more years of education (*R*^2^ = 0.18, *b* = 0.14, 95%CI [0.004, 0.277], *z* = 2.02, *p* = 0.04). Subgroup analysis also revealed measure type to significantly moderate both cognitive empathy, *Q*(5) = 34.80, *p* < 0.001 and empathic concern, *Q*(2) = 8.07, *p* = 0.02 (see Table 5). Specifically, larger effect sizes were obtained with the IRI, BES and HES for cognitive empathy (*g* =  − 0.56 to − 1.58) and with the IRI and SEQ for empathic concern (*g* =  − 0.30 to − 1.12). In addition, location of injury contributed significant variance to empathic concern, with larger effect sizes for insular lesions (*g* =  − 1.55) compared to other brain regions (*g* = 0.25–0.60). However, no significant effects were found for injury type (e.g. stroke, TBI) in any component of empathy.

**Table 4:** Summary of meta-regression analysis for empathy effect sizes

			95%CI			
Moderator	*k*	*b*	Lower	Upper	*Z*	*p*
Affective empathy						
Sex (%male)	14*	− 0.49	− 1.29	0.31	− 1.20	0.23
Age	12	− 0.01	− 0.06	0.03	− 0.61	0.54
Years of education	9	0.21	− 0.02	0.44	1.83	0.07
Time since injury	9	0.02	0.01	0.04	2.45	0.01*
Injury severity						
Post-traumatic amnesia	5	0.01	− 0.01	0.03	0.61	0.54
Glasgow Coma Scale	4	− 0.13	− 0.30	0.05	− 1.42	0.16
Cognitive empathy						
Sex (%male)	26*	− 0.31	− 0.75	0.12	− 1.42	0.16
Age	19	0.01	− 0.01	0.03	0.82	0.41
Years of education	17	− 0.02	− 0.15	0.11	− 0.25	0.80
Time since injury	12	0.01	− 0.03	0.05	0.43	0.67
Empathic concern						
Sex (%male)	25*	− 0.11	− 0.52	0.29	− 0.55	0.58
Age	17	− 0.01	− 0.03	0.01	− 0.91	0.36
Years of education	13	0.14	0.00	0.28	2.02	0.04*
Time since injury	12	< 0.01	− 0.09	0.07	− 0.21	0.83
Personal distress						
Sex (%male)	14*	0.04	− 0.64	0.71	0.11	0.91
Age	10	0.01	− 0.03	0.04	0.34	0.74
Years of education	9	0.21	− 0.06	0.48	1.55	0.12
Time since injury	8	0.08	− 0.05	0.23	1.20	0.23

*k* number of effect sizes, *b* unstandardised beta-weight, *CI* confidence intervals, *p* level of significance

*Higher *k* in these analyses reflects empathy effect sizes being entered for males and females separately (where available). Moderators were tested where there were four or more studies reporting moderator data

**Table 5:** Summary of subgroup analysis

Domain	*k*	*k*	*SE*	95%CI	*z*	*p*	Heterogeneity analysis	
							*Q*(*df*)	*p*
Affective empathy								
Measure type							1.90(4)	0.76
BEES	5	− 0.39	0.18	− 0.73, − 0.04	− 2.17	0.03*		
BES affective + BEES^a^	1	− 0.60	0.46	− 1.50, 0.31	− 1.29	0.20		
EQ affective	1	− 0.40	0.41	− 1.21, 0.40	− 1.98	0.33		
QCAE affective	1	− 0.01	0.42	− 0.83, 0.82	− 0.02	0.99		
QMEE	4	− 0.62	0.23	− 1.07, − 0.17	− 2.70	0.01**		
Cognitive empathy								
Measure type							34.80(5)	< 0.001***
IRI (PT)	13	− 0.56	0.07	− 0.69, − 0.42	− 8.20	< 0.001***		
IRI (FS + PT)	1	− 1.14	0.29	− 1.71, − 0.57	− 3.94	< 0.001***		
BES cognitive	1	− 1.24	0.35	− 1.94, − 0.55	− 3.51	< 0.001***		
EQ cognitive	1	− 0.36	0.22	− 0.79, 0.07	− 1.63	0.10		
HES	2	− 1.58	0.22	− 2.01, − 1.16	− 7.31	< 0.001***		
QCAE cognitive + IRI(PT)^a^	1	− 0.09	0.21	− 0.50, 0.32	− 0.42	0.68		
Injury type							5.16(4)	0.27
TBI	7	− 0.65	0.16	− 0.96, − 0.34	− 4.13	< 0.001***		
Resection	1	− 0.66	0.44	− 1.52, 0.21	− 1.50	0.16		
Stroke	4	− 0.55	0.21	− 0.96, − 0.14	− 2.63	0.01**		
Tumour	2	− 0.28	0.29	− 0.84, 0.27	− 1.00	0.32		
Other/mixed	5	− 1.00	0.19	− 1.37, − 0.63	− 5.24	< 0.001***		
Lesion location							8.05(4)	0.09
Frontal	4	− 1.43	0.31	− 2.05, − 0.82	− 4.56	< 0.001***		
Insular	3	− 0.80	0.35	− 1.50, − 0.11	− 2.28	0.02*		
Temporal	1	− 0.38	0.63	− 1.63, 0.86	− 0.60	0.55		
Posterior	3	− 0.17	0.35	− 0.85, 0.51	− 0.49	0.62		
Other/mixed	13	− 0.71	0.16	− 1.01, − 0.40	− 4.55	< 0.001***		
Empathic concern								
Measure type							8.07(2)	0.02*
IRI (EC subscale)	14	− 0.30	0.13	− 0.56, − 0.04	− 2.25	0.02*		
SEQ	1	− 1.12	0.26	− 1.63, − 0.62	− 4.35	< 0.001***		
TEQ	2	− 0.56	0.40	− 1.33, 0.22	− 1.41	0.16		
Injury type							4.41(4)	0.35
TBI	6	− 0.19	0.26	− 0.41, 0.02	− 1.78	0.08		
Resection	2	− 0.12	0.22	− 0.56, 0.31	− 0.56	0.57		
Stroke	5	− 0.76	0.44	− 1.62, 0.11	− 1.72	0.09		
Tumour	2	− 0.16	0.26	− 0.67, 0.35	− 0.63	0.53		
Other/mixed	2	− 0.78	0.34	− 1.45, − 0.11	− 2.29	0.02*		
Lesion location							12.04(4)	0.02*
Frontal	4	− 0.47	0.28	− 1.02, 0.07	− 1.70	0.09		
Insular	3	− 1.55	0.34	− 2.22, − 0.88	− 4.52	< 0.001***		
Temporal	2	− 0.60	0.41	− 1.40, 0.21	− 1.46	0.15		
Posterior	2	− 0.33	0.41	− 1.13, 0.46	− 0.82	0.41		
Other/mixed	10	− 0.25	0.16	− 0.56, 0.05	− 1.62	0.11		
Personal distress								
Injury type							5.57(4)	0.23
TBI	4	0.02	0.29	− 0.55, 0.58	0.06	0.95		
Resection	1	− 0.18	0.29	− 0.74, 0.38	− 0.64	0.52		
Stroke	3	− 0.44	0.72	− 1.86, 0.98	− 0.61	0.55		
Tumour	1	0.49	0.22	0.07, 0.92	2.27	0.02*		
Other/mixed	1	− 0.11	0.25	− 0.59, 0.38	− 0.44	0.66		

Moderators were tested where there were four or more studies reporting moderator data

*k* number of effect sizes, *g* standardised mean difference (Hedges’ *g*), *SE* standard error, *CI* confidence intervals, *p* level of significance, TBI traumatic brain injury

^a^The mean effect size of two subscales

### Publication Bias

As can be seen in Table 2, Rosenthal’s (1979) Fail-Safe *N* analysis suggested that effect sizes for affective empathy, cognitive empathy and empathic concern were robust to publication bias, as their respective Fail-Safe *N*s exceeded 5*n* + 10. Additionally, funnel plots did not show any clear visual evidence of small sample bias (see Fig. S3, Supplementary material) and Egger’s test of asymmetry of funnel plots was non-significant for all effect sizes.

## Discussion

Here, we present the first meta-analytic review of empathic functioning after acquired brain injury. Compared to matched, healthy controls, ABI in adults was found to be associated with moderate self-reported deficits in both cognitive (*g* = −0.68) and affective (*g* = −0.43) empathy and small-to-moderate deficits in empathic concern (e.g., sympathy, *g* =  − 0.38). The prevalence of empathy deficits after ABI was estimated to be 35.0% for affective empathy (versus 19.7% controls), 31.4% for cognitive empathy (versus 11.8% controls), and 21.6% for empathic concern (versus 14.8% controls). However, the prevalence of low personal distress did not differ significantly between ABI and control participants. We found evidence of large variation in the strength of effects, which is unsurprising given the marked heterogeneity of ABI. These findings align with a growing body of literature suggesting that empathy is commonly affected after brain injury. We extend this literature by providing a more precise estimate of prevalence and effect size across the different facets of empathy.

### Low Empathy in ABI and its Impact on Socio-Emotional Functioning

We found a global empathy deficit in ABI, that is, a reduced ability or propensity to feel others’ emotions (affective empathy), as well as recognize and understand those emotions (cognitive empathy). Broadly, these results align with evidence that brain injury can affect both early and later stages of emotional processing. Whilst affective empathy occurs via early “bottom-up” perceptual and mimicry mechanisms, cognitive empathy recruits “top-down” processes of cognitive control later in the processing time course (Thompson et al., 2019). Similarly, numerous sources point to alterations in early affective processing in TBI (e.g. reduced facial responding, lower autonomic arousal), as well as impaired inhibitory control in both TBI and stroke (Carota et al., 2002; McDonald & Genova, 2021). Of note, we observed a significantly stronger effect size for cognitive empathy, suggesting that this aspect of empathy may be most impaired after ABI. Given the effortful processing involved in cognitive empathy, this ability might be more vulnerable to neurological damage. Indeed, reviews of several neurological conditions have noted larger deficits in cognitive empathy compared to other empathy components, including Alzheimer’s disease (Demichelis et al., 2020), Parkinson’s dementia (Coundouris et al., 2020), multiple sclerosis (Lin et al., 2021b) and epilepsy (Wang et al., 2022). However, it is noted that few studies in this review investigated both affective and cognitive components concurrently within the same cohort (*k* = 6). Given the substantial clinical heterogeneity between studies, caution is needed when directly comparing cognitive and affective empathy effect sizes. Future research should therefore measure both components within the same sample to determine whether the same pattern arises.

Since affective and cognitive empathy processes work in concert to promote interpersonal bonding and prosocial behaviour (Weisz & Cikara, 2021), the current findings suggest that some people with ABI who have empathy deficits might experience difficulties connecting to others. Preliminary evidence appears to support this: perspective-taking (i.e. cognitive empathy) deficits have been shown to predict interpersonal and communication problems after ABI (Saxton et al., 2013), as well as self-reported loneliness (Adams et al., 2020) and lower social competence in the workplace (Yeates et al., 2016). Researchers have postulated that difficulty considering others’ perspectives might partially contribute to certain behaviours such as limited understanding of others’ needs and behaviour, leading to misinterpretations, poor listening skills and inappropriate responses (Tousignant et al., 2018; Williams et al., 2020), known in general populations to negatively influence relationship quality and satisfaction (Cahill et al., 2020; Walker et al., 2024). Thus, based on some initial findings, empathy difficulties may play a role in post-injury functioning across a range of social contexts, placing the individual at greater risk of social isolation. There is also a higher prevalence of ABI in prison populations (Matheson et al., 2020), with lack of empathy potentially contributing to offending behaviour (Van Langen et al., 2014). However, these relationships remain speculative. Empathy deficits rarely occur in isolation after ABI (Henry et al., 2016), and it is still unclear how they specifically impact social functioning compared to the broad spectrum of neurobehavioural sequelae.

### Comparisons with Other Meta-Analyses of Emotional Processing After ABI

Compared to our results, meta-analyses of broader, related emotional processes in brain injury have typically shown larger effect sizes, such as emotion recognition deficits in TBI patients (*g* =  − 0.79, Murphy et al., 2022) and self-reported alexithymia (Fynn et al., 2021, *g* =  − 1.00) in ABI. Furthermore, a recent meta-analysis by Lin et al. (2021a) found that adults with TBI displayed very large deficits in affective Theory of Mind (ToM, *g* =  − 1.24), a construct often regarded as analogous to cognitive empathy (Schurz et al., 2021). It may be that objective tasks are simply more sensitive measures of empathy deficits. Self-report tools (like the ones used in our meta-analysis) depend on participants’ insight, motivation and memory to report accurately on their behaviour, domains known to be impacted by brain injury (e.g. Gasquoine, 2016). Indeed, underreporting of empathy deficits has been consistently noted in brain-injured patients compared to the evaluations of friends and family (Bramham et al., 2009; Burridge et al., 2007; Jorna et al., 2021; Wells et al., 2005; Zupan et al., 2018). Although Fynn et al. (2021) found large self-reported deficits for alexithymia in ABI patients, it may be that empathy is particularly vulnerable to underreporting, given its high social desirability (Laurent & Hodges, 2009; Sassenrath, 2020). Nonetheless, some ABI research has reported moderate-to-strong correlations between self-reported empathy and objective assessment measures of empathy-related responding. Specifically, lower empathy has been linked to reduced physiological responding to angry facial expressions (*r* = 0.410, *p* = 0.006; de Sousa et al., 2011), hypoarousal to pleasant pictures (*r* = 0.635, *p* < 0.008; de Sousa et al., 2010) and poorer performance on Theory of Mind tasks (*r* = −0.509, *p* < 0.001; Shamay-Tsoory et al., 2005). Whilst research is yet to report inter-rater reliability for empathy specifically, a significant correlation was recently reported for self- versus informant-rated social cognition in an ABI sample (*r* = 0.47, *n* = 49, *p* < 0.05; Cattran et al., 2016).

### Empathic Concern and Personal Distress

In addition to cognitive and affective empathy, we also examined empathy-related responding: empathic concern (e.g. sympathy) and personal distress. Small-medium deficits in empathic concern were found in individuals with ABI versus healthy controls (*g* =  − 0.38), suggesting that on average, individuals with brain injury are less likely to feel sympathy and concern for others. Empathic concern activates neural networks involved in reward processing, including the ventral striatum, insula and medial orbitofrontal cortex. These regions are known to be vulnerable to injury from TBI (Shah et al., 2012) and stroke (Widmer et al., 2019). Low empathic concern could equally arise from the downstream effects of impaired (cognitive and affective) empathy, as a certain level of empathic arousal may be needed to provoke concern for another’s wellbeing (Decety, 2010). Regardless of the mechanism, lower levels of emotional warmth, concern and other-oriented motivation are likely to be problematic for survivors’ interpersonal relationships, since empathic concern is thought to be crucial in maintaining friendships and close relationships (Kardos et al., 2017; Morelli et al., 2017). Partners of adults with brain injury are particularly at risk of feeling emotionally disconnected from their spouse over time (Yeates et al., 2013) and lower empathic concern in neurological patients has been linked to relationship dissatisfaction (Burridge et al., 2007) and caregiver burnout (Martinez et al., 2018).

Overall, ABI participants were not found to differ from controls on personal distress — the tendency to experience self-focused, negative arousal in response to others’ distress. This result is somewhat surprising, as our results indicated less emotion sharing (i.e. affective empathy) overall in adults with brain injury compared to non-brain-injured peers. Since most personal distress items measure coping in emergency situations (e.g. “I tend to lose control during emergencies”), it may be that the emotional intensity of such situations is sufficiently strong to generate an empathic response. In line with this, Holtmann et al. (2023) recently reported affective empathy deficits in insular lesion patients in response to low-intensity, but not high-intensity negative emotions. Alternatively, there may be times where personal distress bypasses emotion sharing altogether, for example feeling panicked in a stressful situation (Eisenberg et al., 2007). We also note that this effect size was associated with the highest level of heterogeneity. Among the individual studies reporting significant results, three reported higher levels of personal distress in ABI versus controls (McDonald et al., 2017; Pertz et al., 2021, 2022), and one lower (Holtmann et al., 2023). Davis (1980) conceptualized the personal distress subscale as a measure of emotional reactivity — the degree to which an individual responds to emotional stimuli (Werner et al., 2007). Emotional reactivity can be diversely impacted after brain injury: it can be dampened after damage to the ‘energization system’ (e.g. apathy; Stuss, 2013) or heightened due to cognitive control deficits (Salas et al., 2018). Further research is needed to disentangle these profiles and their impact on empathy.

### Moderating Factors

We found no impact of sex on empathy effect sizes, suggesting that brain injury does not affect males’ empathy abilities differently to females. Conversely, Zupan et al. (2018) found equivalent levels of self-reported cognitive empathy and empathic concern in males versus females with severe TBI (*N* = 160), suggesting that the typically observed female advantage for empathy may disappear after brain injury. However, since we were only able to obtain separate male and female data for nine primary studies, we may have lacked power to detect a moderating effect. Future research may consider reporting empathy data for males and females separately, to further explore this question.

Our analyses did reveal several demographic, injury and measurement characteristics to be significant moderators of empathy. First, greater educational attainment was found to predict self-reported higher empathic concern. To our knowledge, this relationship has not previously been reported in ABI, although our finding aligns with prior research in non-clinical samples (Sommerlad et al., 2021; Yaghoubi Jami et al., 2021). A commonly used proxy of cognitive reserve (Chapko et al., 2017), education has been identified as a key protective factor against cognitive decline in ABI, predicting better prognosis and rehabilitation outcomes (for reviews, see Contador et al., 2023; Nunes & Silva Nunes, 2021). It may be useful for future studies to explore whether cognitive reserve plays a role in preserving empathy ability after brain injury, since higher education has been found to mitigate age-related decreases in empathy in healthy older adults (Gutiérrez-Cobo et al., 2023).

Second, meta-regression revealed a positive association between time since injury and affective empathy scores in ABI participants, in contrast to prior work reporting no relationship between these variables (Osborne-Crowley et al., 2020; Williams & Wood, 2010; Wood & Williams, 2008). Longitudinal research has documented a degree of spontaneous recovery in cognitive and emotional functioning after brain injury, particularly in the first-year post-injury (Carmichael et al., 2023; Ponsford et al., 2014; Rabinowitz et al., 2018). However, these studies noted great variability in individuals’ trajectories of recovery, and other research has shown stability of social cognitive deficits over time (Milders et al., 2006). In addition, since there were less than 10 studies in this meta-regression, this result should be interpreted with caution (Higgins et al., 2024). Finally, given the cross-sectional nature of our data, causality cannot be inferred. Rather, longitudinal designs are needed to determine whether affective empathy deficits may improve over time.

Third, lesion location was found to significantly impact empathic concern deficits for ABI patients relative to controls. Specifically, insular lesions were associated with large deficits in empathic concern (*g* =  − 1.55) in individuals with ABI, compared to other brain regions (*g* = 0.25–0.60). Insular activation has been consistently observed in fMRI studies of compassion tasks and self-reported empathic concern (for reviews, see Kim et al., 2020; Novak et al., 2022) and is thought to play a key role in the integration of sensory, emotional and social information (Damasio, 2000). Neither lesion location (*p* = 0.09) nor injury type (*p* = 0.27) significantly moderated effect sizes for cognitive empathy, although the high proportion of TBI cases in the mixed aetiology samples limited our ability to compare effect sizes across injury locations and types. Finally, measure type was an additional significant source of variance for cognitive empathy and empathic concern, with effect sizes differing up to 1.4 standard deviations depending on the measure used.

### Clinical Implications

Our results highlight the importance of assessing empathy in clinical settings and providing psychoeducation to patients and their families about empathy-related changes after brain injury. Moreover, it underscores the importance of using questionnaire measures that assess both cognitive and affective empathy, since impairments were observed across both components. However, we caution against relying solely on a global empathy score. Empathy profiles may differ considerably at an individual level, since empathy components can be selectively impaired depending on the location of injury (Campanella et al., 2022; Shamay-Tsoory et al., 2004, 2009). Our research suggests that evidence-based treatments for improving empathy ability are urgently needed to help patients and families address these challenges. Furthermore, the current results suggest that interventions should consider targeting both affective and cognitive components. Recent trials of interventions targeting perspective-taking, emotion recognition and emotion regulation in brain-injured patients and their partners have shown promising results in improving empathic behaviour and partner communication and relationship quality (Backhaus et al., 2019; Martín-Rodríguez & León-Carrión, 2010; Westerhof-Evers et al., 2017). However, more trials are needed to determine their generalizability, pinpoint the mechanisms of change and map the trajectory of deficits over time. Finally, given the high prevalence of ABI among people who experience incarceration (Matheson et al., 2020), further exploration of the association between empathy and offending behaviour in offenders with ABI would be a valuable research direction.

### Limitations and Recommendations for Future Research

Our findings must be considered in the context of certain limitations. First and foremost, these relate to empathy measurement. Diverse conceptualisations of empathy abound and researchers have increasingly argued the need for consistent, clear-cut definitions to provide clarity about underlying constructs (Decety & Cowell, 2014; Hall & Schwartz, 2019; Stietz et al., 2019). Whilst it has been common practice to measure empathic concern and personal distress as proxies of affective empathy, the validity of this approach has been questioned on the basis that these are distinct psychological and neurological processes (Decety, 2010) with different interpersonal and behavioural outcomes (Lamm et al., 2007; Weisz & Cikara, 2021). Therefore, a strength of this study was the attempt to delineate the various empathy-related components. Moreover, our differing results for each facet of empathy strengthen the rationale for this approach. Nonetheless, some threats to validity were noted. Firstly, some measures contained elements of several components. For example, whilst the BEES mostly reflects emotion sharing, it also contains several items relating to empathic concern (e.g. ‘I get a strong urge to help when I see someone in distress’) and personal distress (e.g. ‘It upsets me to see someone being mistreated’). Nonetheless, a qualitative review of item content suggested that the majority of items in each scale (e.g. > 65%) measured the construct of interest, with relatively low overlap with other components. Secondly, some tools may not have fully captured the relevant construct, such as the *perspective-taking* subscale of the IRI assessing one aspect of cognitive empathy. More recent measures (e.g., QCAE, BES) include items about the ability to notice, recognise and understand others’ emotions, which more closely reflects current definitions of cognitive empathy (Decety & Jackson, 2004). Additionally, the recently introduced Perth Empathy Scale (PES; Brett et al., 2023) has demonstrated that people’s empathy abilities (particularly affective empathy) can differ in a valence-specific manner, depending on whether the emotion in question is negative or positive. Therefore, we encourage researchers in future studies to select empathy questionnaires that measure cognitive and affective empathy comprehensively and in line with recent conceptualisations of empathy. It is also important that these measures become standardized over time to decrease the likelihood of discrepant findings. Lastly, insight deficits resulting from brain injury may affect validity of self-report measures and lead to underreporting of challenges. As such, it is recommended to additionally use informant reports where possible.

Although we were able to explore the impact of certain demographic factors and injury characteristics on empathy, high levels of missing data limited moderator analysis across all components of empathy. The number of included studies in each moderator analysis was also relatively low (e.g. generally < 20); therefore, power constraints may have reduced our ability to detect moderator effects. More research in this area would enable exploration of how empathy levels may differ across factors such as injury type and time since injury.

Prevalence estimates for empathy deficits were based on simulated data, an approach that assumes normal distribution of empathy scores. We consider the latter to be a reasonable assumption, since empathy is reported to be normally distributed in the general population (Baron‐Cohen et al., 2001; Brett et al., 2023). Moreover, all available raw empathy data were found to be approximately normally distributed. However, the true distribution shape of empathy scores in the ABI population is unknown and any deviations from normality would impact the accuracy of our results. Thus, it would be preferable to base future estimates on real data. To this end, we encourage researchers to make prevalence data available or report on the distribution shape of empathy scores.

Finally, our quality assessment revealed significant bias in patient selection. The primary issues identified were unclear inclusion criteria, clinical and control groups differing on key demographic variables and reliance on convenience-based sampling methods. We also note that approximately a third of the study set consisted of lesion studies, which recruited participants with specific injuries thought to be associated with empathy deficits (e.g. no. 2, no. 3, no. 4, no. 10, no. 14, no. 22, no. 23, no. 24, no. 29).These studies most frequently assessed cognitive empathy and empathic concern; hence, our estimates for these components may have been inflated by this. Overall, the identified issues were unsurprising given the inherent challenges of recruitment in neurological research (Newberry et al., 2010). However, caution is needed in applying the results of this analysis to the ABI population as a whole.

## Conclusions

Empathy is integral to interpersonal functioning, yet has only been examined relatively recently within the ABI literature. According to our analysis, approximately one third of adults with an ABI endorse difficulties sharing and understanding others’ emotions. This is a considerable proportion of the ABI population and may yet be an underestimate, since individuals with brain injury have been shown to understate their challenges. Our results strengthen the rationale for routine assessment of empathy in clinical settings and the development of targeted interventions. Further research is needed to explore the impacts of sociodemographic and injury-related factors on empathy, to help determine who may be most at risk of impairment.

## Supplementary Information

Below is the link to the electronic supplementary material.

ESM 1 (DOCX 115 KB)

ESM 2 (DOCX 33.0 KB)

## Data Availability

The data used to support the findings of this study are available from the corresponding author upon request.
