# Supplementary material for: Empathy in Adults with Acquired Brain Injury: a Systematic Review and Meta-Analysis
Source: Neuropsychol Rev. 2025 Jun 18;36(2):160–83. doi: 10.1007/s11065-025-09667-5 (PMC13388439; doi:10.1007/s11065-025-09667-5)
Supplement: Supplementary file 1 — (DOCX 115 KB) [file 11065_2025_9667_MOESM1_ESM.docx]

***Supplementary Information***

**Table S1**

*Details of Search Strategy*

| **Database** | **Search Query** | |
| --- | --- | --- |
| Medline, PsycInfo | #1 | (Empath* or interpersonal reactivity or emotion* contagion or experience sharing or theory of mind or mentali?ing or perspective taking).mp. or social cognition.tw. [mp=title, abstract, heading word, table of contents, key concepts, original title, tests & measures, mesh word] |
|  | #2 | ((brain or head or craniocerebral or cranial* or cerebr*) adj2 (injur* or trauma* or incident or accident or damage or concussi* or "brain ischemi*" or stroke or "diffuse axonal injur*" or tbi or abi or abd)).tw. |
|  | #3 | exp "theory of mind"/ |
|  | #4 | exp empathy/ |
|  | #5 | exp social cognition/ |
|  | #6 | 1 or 3 or 4 or 5 |
|  | #7 | exp Brain Injuries/ |
|  | #8 | exp Brain Damage/ |
|  | #9 | 2 or 7 or 8 |
|  | #10 | 6 and 9 |
| Scopus | Advanced query( TITLE-ABS-KEY ( empath* OR "interpersonal reactivity" OR "emotion* contagion" OR "experience sharing" OR "theory of mind" OR mentalizing OR "perspective taking" OR "social cogniti*" ) AND ( ( brain OR head OR craniocerebral OR cranial OR cerebr* ) PRE/2 ( injur* OR trauma* OR incident OR accident OR damage OR concussi* OR "brain ischemi*" OR stroke OR "diffuse axonal injur*" OR tbi OR abi OR abd ) ) ) AND ( LIMIT-TO ( DOCTYPE , "ar" ) OR LIMIT-TO ( DOCTYPE , "re" ) OR LIMIT-TO ( DOCTYPE , "cp" ) ) AND ( LIMIT-TO ( LANGUAGE , "English" ) ) AND ( EXCLUDE ( SUBJAREA , "VETE" ) OR EXCLUDE ( SUBJAREA , "EART" ) OR EXCLUDE ( SUBJAREA , "ECON" ) OR EXCLUDE ( SUBJAREA , "ENER" ) OR EXCLUDE ( SUBJAREA , "DECI" ) OR EXCLUDE ( SUBJAREA , "MATE" ) OR EXCLUDE ( SUBJAREA , "CENG" ) OR EXCLUDE ( SUBJAREA , "CHEM" ) OR EXCLUDE ( SUBJAREA , "PHYS" ) OR EXCLUDE ( SUBJAREA , "IMMU" ) OR EXCLUDE ( SUBJAREA , "MATH" ) OR EXCLUDE ( SUBJAREA , "BUSI" ) OR EXCLUDE ( SUBJAREA , "ENGI" ) OR EXCLUDE ( SUBJAREA , "ENVI" ) OR EXCLUDE ( SUBJAREA , "AGRI" ) OR EXCLUDE ( SUBJAREA , "PHAR" ) OR EXCLUDE ( SUBJAREA , "COMP" ) ) | |
| ProQuest | **(abstract(empath* OR "interpersonal reactivity" OR "emotion* contagion" OR "experience sharing" OR "theory of mind" OR mentali?ing OR "perspective taking" OR "social cogniti*) OR title(empath* OR " interpersonal reactivity " OR " emotion* contagion " OR " experience sharing " OR " theory of mind " OR mentali?ing OR " perspective taking " OR " social cogniti*)) AND abstract((brain OR head OR craniocerebral OR cranial OR cerebr*) NEAR/2 (injur* OR trauma* OR incident OR accident OR damage OR concussi* OR "brain ischemi*" OR stroke OR "diffuse axonal injur*" OR tbi OR abi OR abd) OR title (brain OR head OR craniocerebral OR cranial OR cerebr*) NEAR/2 (injur* OR trauma* OR incident OR accident OR damage OR concussi* OR "brain ischemi*" OR stroke OR "diffuse axonal injur*" OR tbi OR abi OR abd))** | |
| Web of Science | (((TS=(empath* OR interpersonal reactivity OR emotion* contagion OR experience sharing OR theory of mind OR mentali?ing OR perspective taking OR social cogniti* )) AND TS=(((brain OR head OR craniocerebral OR cranial OR cerebr*))) AND TS=((injur* OR trauma* OR incident OR accident OR damage OR concussi* OR brain ischemi* OR stroke OR diffuse axonal injur* OR tbi OR abi OR abd)))) NOT (DT==("BIOGRAPHICAL ITEM" OR "CORRECTION" OR "BOOK REVIEW" OR "MEETING ABSTRACT" OR "MEETING" OR"NOTE" OR "BOOK CHAPTER" OR “REVIEW”)) and English (Languages) and Neurosciences Neurology or Psychology or Rehabilitation or Psychiatry or Behavioral Sciences or General Internal Medicine or Surgery or Research Experimental Medicine or Oncology (Research Areas) | |

**Table S2**

*Empathy Measures and Their Corresponding Components*

| **Empathy Group** | **Definition** | **Empathy Measures** |
| --- | --- | --- |
| **Overall Empathy** | Feeling, sharing and understanding another’s emotional experiences, whilst maintaining self-other awareness. Includes both cognitive and affective processes. | QCAE, BES, EQ |
| **Affective Empathy** | The process of sharing another’s emotion(s) | QCAE (Affective subscale), BES (Affective subscale), BEES, QMEE/EEQ, EQ (Affective subscale) |
| **Cognitive Empathy** | The process of recognising and understanding another’s emotion(s) | QCAE (Cognitive subscale), BES (cognitive subscale), IRI (Perspective-taking subscale; Perspective-taking + Fantasy subscale composite score), EQ (Cognitive subscale), HES |
| **Empathic Concern** | Feelings of sympathy and compassion for another | IRI (Empathic Concern subscale), TEQ, SEQ – empathy subscale |
| **Personal Distress** | An aversive state of negative emotional arousal elicited by others’ distress | IRI (Personal Distress subscale) |

*Note.* QCAE = Questionnaire of Cognitive and Affective Empathy; BES = Basic Empathy Scale; EQ = Empathy Quotient; IRI = Interpersonal Reactivity Index; HES = Hogan Empathy Scale; BEES = Balanced Emotional Empathy Scale; QMEE = Questionnaire Measure of Emotional Empathy; EEQ = Emotional Empathy Questionnaire; TEQ = Toronto Empathy Questionnaire; SEQ = Social Emotional Questionnaire

**Table S3**

*Summary of QUADAS-2 Assessment of Included Studies*

|  |  | **Risk of Bias** | | | |  | **Applicability Concerns** | | |
| --- | --- | --- | --- | --- | --- | --- | --- | --- | --- |
| # |  | **Patient Selection** | **Index Test** | **Reference Standard** | **Flow and Timing** |  | **Patient Selection** | **Index Test** | **Reference Standard** |
| 1 | Adams et al. (2021) | Low | Low | Unclear | Low |  | Low | Low | Low |
| 2 | Boucher et al. (2015) | Low | Low | Low | Unclear |  | Low | Low | Low |
| 3 | Bramham et al. (2009) | Low | Low | Low | Low |  | Low | Low | Low |
| 4 | Chen et al. (2016) | Low | Low | Low | Low |  | Low | Low | Low |
| 5 | Darling (2001) | Unclear | Low | Unclear | Unclear |  | Low | Low | Unclear |
| 6 | Driscoll & Krueger (2012) | Low | Low | Unclear | Low |  | Low | Low | Unclear |
| 7 | Gallant & Good (2020) | High | Low | High | Unclear |  | Unclear | Low | Unclear |
| 8 | Goebel et al. (2018) | Low | Low | Low | Low |  | Low | Low | Low |
| 9 | Grattan & Eslinger (1989) | Low | Low | Low | Low |  | Low | Low | Low |
| 10 | Holtmann et al. (2023) | Unclear | Low | Unclear | Low |  | Low | Low | Unclear |
| 11 | Kgolo et al. (2021) | Low | Low | Unclear | Low |  | Low | Low | Low |
| 12 | McDonald et al. (2017) | Low | Low | Low | Unclear |  | Low | Low | Low |
| 13 | Milders et al. (2003) | Unclear | Low | Low | Unclear |  | Low | Low | Low |
| 14 | Minga (2015) | Unclear | Low | Unclear | Low |  | Low | Low | Low |
| 15 | Muller et al. (2010) | Unclear | Low | Low | Low |  | Low | Low | Low |
| 16 | Neumann et al. (2014) | Unclear | Low | Low | Unclear |  | Low | Low | Low |
| 17 | Neumann et al. (2021) | Unclear | Low | Low | Low |  | Low | Low | Low |
| 18 | Nijsse et al. (2019b) | Low | Low | Low | Low |  | Low | Low | Low |
| 19 | Paterson (2011) | Low | Low | Low | Unclear |  | Low | Low | Low |
| 20 | Pertz et al. (2021) | Low | Low | Low | Low |  | Low | Low | Low |
| 21 | Pertz et al. (2022) | Low | Low | Unclear | Low |  | Low | Low | Low |
| 22 | Sawczak et al. (2022) | Low | Low | Low | Unclear |  | Low | Low | Low |
| 23 | Shamay-Tsoory et al. (2004) | Low | Low | Unclear | Low |  | Low | Unclear | Unclear |
| 24 | Shamay-Tsoory et al. (2009) | Unclear | Low | Unclear | Unclear |  | Low | Low | Low |
| 25 | Spikman et al. (2012) | Low | Low | Low | Low |  | Low | Low | Low |
| 26 | Williams & Wood (2010) | Low | Low | Low | Low |  | Low | Low | Low |
| 27 | Wood & Williams (2008) | Low | Low | Low | Low |  | Low | Low | Low |
| 28 | Yeh & Tsai (2014) | Low | Low | Unclear | Low |  | Low | Low | Low |
| 29 | Yeh et al. (2015) | Unclear | Low | Low | Low |  | Low | Low | Low |

**Fig. S1**

*Bar Charts for QUADAS-2 Analysis*


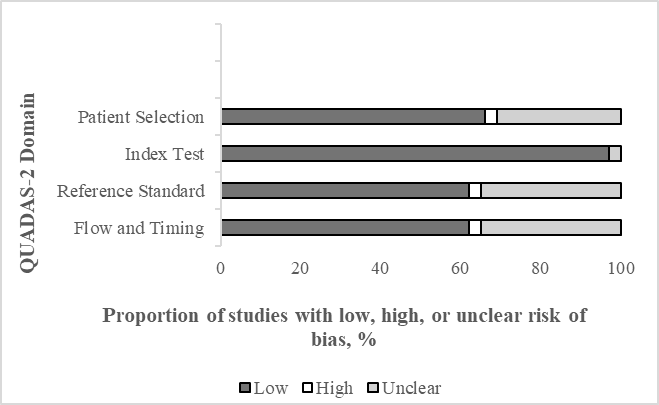


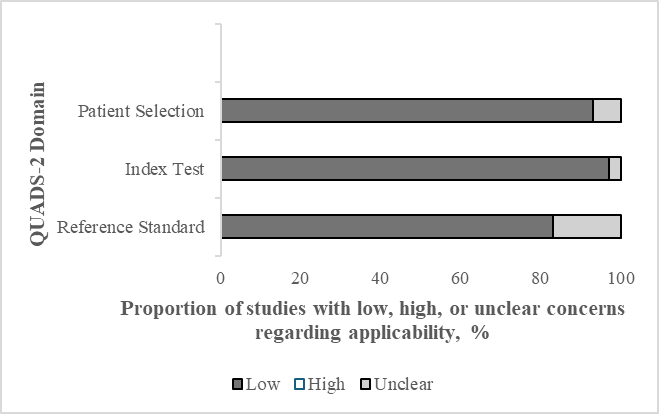


*Note.* QUADAS-2; Quality Assessment of Diagnostic Accuracy Studies 2

**Fig. S2**

*Forest Plots of Standardized Mean Difference in Empathy Between ABI Participants and Controls*

Panel A: Affective Empathy

Panel B: Cognitive Empathy

Panel C: Empathic Concern

Panel D: Personal Distress

Note. The boxes represent Hedges’ *g* for each study. The horizontal lines show 95% confidence intervals. The diamond below the studies denotes the overall pooled effect from the included studies.

**Fig. S3**

*Funnel Plots Based on Standardized Mean Difference (g) in Empathy Between ABI Sample and Controls*

Panel A: Affective Empathy Panel B: Cognitive Empathy

Panel C: Empathic Concern Panel D: Personal Distress

*Note.* The dashed lines indicate the funnel. Each circle represents an individual study

**Supplement Box 1**

*Deviations from Pre-Registered Protocol*

| 1. Change of risk of bias tool - from Johanna Briggs Institute Critical Appraisal Tools (Johanna Briggs Institute, 2017) in protocol to Quality Assessment of Diagnostic Accuracy Studies-2 framework (QUADAS-2; Whiting, 2011) in the systematic review. The latter has a greater emphasis on study design and execution and was judged more appropriate for this review. 2. Addition of years of education, lesion location and time since injury as moderators in the systematic review, since these factors have been previously found to moderate aspects of social cognition (Herbet et al., 2015; Lin et al., 2021b; Nijsse et al., 2019b; Shamay-Tsoory et al., 2009; Wang et al., 2014). 3. Sensitivity analyses were conducted in the systematic review to validate prevalence estimates. |
| --- |
